# Lean Body Mass as a Predictor of Levothyroxine Requirement in Primary Hypothyroidism as Compared to Actual Body Weight

**DOI:** 10.1210/jendso/bvaf159

**Published:** 2025-10-16

**Authors:** Damacharlla Venkateswarlu, Manjunath Goroshi, Vanishri Ganakumar, Vikrant Ghatnatti, Sruthi Kotla

**Affiliations:** Department of Endocrinology, Jawaharlal Nehru Medical College, KAHER, Belagavi 590010, Karnataka, India; Department of Endocrinology, Jawaharlal Nehru Medical College, KAHER, Belagavi 590010, Karnataka, India; Department of Endocrinology, Jawaharlal Nehru Medical College, KAHER, Belagavi 590010, Karnataka, India; Department of Endocrinology, Jawaharlal Nehru Medical College, KAHER, Belagavi 590010, Karnataka, India; Department of Endocrinology, Jawaharlal Nehru Medical College, KAHER, Belagavi 590010, Karnataka, India

**Keywords:** primary hypothyroidism, levothyroxine, lean body mass, actual body weight, ideal body weight

## Abstract

**Context:**

Levothyroxine (LT4) therapy for hypothyroidism is traditionally dosed at 1.6 μg/kg of actual body weight (ABW). However, ABW-based dosing often fails to proportionally adjust for increasing body weight. Ideal body weight (IBW) and lean body mass (LBM) have been proposed as alternatives, but data on these parameters on the larger hypothyroid population are lacking, particularly in Hashimoto thyroiditis.

**Objective:**

This study evaluates LBM- and IBW-based LT4 dosing and examines variability in ABW-based dosing across age, body mass index (BMI), and menopausal status.

**Methods:**

This cross-sectional study analyzed 720 patients with primary hypothyroidism on stable LT4 doses and in a euthyroid state for ≥6 months. ABW, BMI, LT4 dose, and thyrotropin (TSH) were recorded. IBW and LBM were calculated using Devine's and Boer's formulas, respectively. LT4 doses per ABW, IBW, and LBM were compared across age, BMI, and menopause.

**Results:**

Daily LT4 dose per kilogram of ABW decreased across BMI categories (18.5-24.9 kg/m^2^: 1.73 ± 0.34 µg/kg, 25-29.9 kg/m^2^: 1.51 ± 0.30 µg/kg, ≥ 30 kg/m^2^: 1.33 ± 0.31 µg/kg; *P* < .001). In contrast, IBW-based dosing increased with BMI (1.92 ± 0.40, 2.09 ± 0.47, 2.25 ± 0.51 µg/kg respectively; *P* < .001), while LBM-based dosing remained consistent (2.37 ± 0.48, 2.37 ± 0.52, 2.35 ± 0.54 µg/kg respectively; *P* = .91). LT4 dose decreased significantly with age (*P* < .001) in ABW-based dosing but showed no significant change with LBM (*P* = .224) or IBW (*P* = .377). ABW-based dosing was significantly lower in postmenopausal patients (*P* < .001), while IBW and LBM-based dosing showed no significant variation.

**Conclusion:**

LT4 dosing based on LBM offers a more consistent approach for managing hypothyroidism. A dose of 2.3 mcg/kg LBM may optimize treatment outcomes.

Primary hypothyroidism is a common endocrine problem we come across in clinical practice. It affects approximately 11% of the Indian population, significantly higher than in the Western countries (2%-4%) [1]. Untreated hypothyroidism can lead to systemic complications, such as cardiovascular, neuromuscular, and metabolic disorders.

In patients diagnosed with hypothyroidism, treatment with levothyroxine (LT4) is often started at a dosage of 1.6 μg/kg of actual body weight (ABW). Studies involving ABW indicate that as body weight rises, the dose of levothyroxine also increases; however, the dose increment was not proportional to the increase in ABW [2-4]. India is experiencing a rapid increase in the prevalence of obesity (40.2%), because of changes in lifestyle habits [5]. Utilizing ABW for treatment may result in overdosing of levothyroxine in obese or overweight patients, which may increase the adverse effects of levothyroxine on the cardiovascular system and bone. Additionally, this approach may lead to excessive financial burden on patients, in the form of more frequent testing and follow-up.

Apart from weight, other parameters like age and menopausal status also determine levothyroxine dosage [6]. Alternatively, dosing based on ideal body weight (IBW) or lean body mass (LBM) have also been used to determine levothyroxine dose. Studies indicate that LBM is a significant factor in determining the daily thyroid hormone requirements for elderly patients [7].

There is a lack of comprehensive studies that incorporate the effects of age and hormonal status, particularly menopause, into LT4 dosing algorithms. Moreover, most of the previous studies on LT4 dosing in primary hypothyroidism based on IBW and LBM have focused on patients who underwent thyroidectomy. There is limited data on LT4 dosing in patients with primary hypothyroidism caused by Hashimoto thyroiditis, which is the most prevalent cause of hypothyroidism. This study was designed to fill these gaps by offering a detailed comparison of LT4 dosing methods, while accounting for critical variables such as body mass index (BMI), age, and menopausal status, providing a more individualized approach to hypothyroidism management.

## Objectives

1. To assess LBM-based and IBW-based dosing of LT4 in different age groups, BMI categories, and menopause status in patients with primary hypothyroidism.
2. To assess the variability of ABW-based LT4 dose depending on age, BMI, and menopause status in patients with primary hypothyroidism.

## Methods

This cross-sectional study was conducted from February 2024 to July 2024 at a tertiary care center in Belagavi, involving adult patients with long-standing (>10 years) overt primary hypothyroidism who were euthyroid (0.5-2.5 mU/L) on stable LT4 dose (≥75 mcg/day) for at least 6 months. Institutional Ethics Committee approval was obtained prior to the initiation of the study. Written informed consent was obtained from all participants before their enrollment. Patients with post-thyroidectomy/post-radioiodine ablation hypothyroidism, central or subclinical hypothyroidism, pregnant women, and those with conditions or medications affecting thyroid hormone absorption were excluded.

### Data Collection

All consecutive patients who agreed to participate and met the specified inclusion and exclusion criteria were included in the study. The information gathered comprised age, gender, height, body weight, menopausal status for female participants, adherence to medication, co-prescription within 4 hours of LT4, and the duration of fasting before taking LT4. Body weight was measured using electronic scales, while height was determined with a stadiometer, ensuring that the head was aligned in the Frankfurt horizontal plane. IBW was calculated (as per Devine's formula) [8]: For men, IBW (in kgs) = 50.0 kg + 0.9 × (height in cm − 152); For women, IBW (in kgs) = 45.5 + 0.9 × (height in cm − 152). LBM was calculated based on the Boer Formula [9]: for males, LBM = 0.407 × Weight + 0.267 × Height − 19.2; for females, LBM = 0.252 × Weight + 0.473 × Height − 48.3. Thyrotropin (thyroid stimulating hormone; TSH) readings from the last 6 months were noted.

Patients were divided into 3 groups depending on their age (18-45 years, 46-65 years, and > 65 years). Depending on BMI, again, patients were divided into 3 groups (18.5-24.9, 25-29.9, and ≥ 30 kg/m^2^). Female patients were divided into 2 groups: premenopausal and postmenopausal.

### Biochemical Analysis

Serum TSH testing was conducted using the chemiluminescence immunoassay technique (Roche Cobas e402), which has a functional sensitivity of 0.005 mIU/L and an established normal range of 0.27-4.2 mIU/L. The intra-assay variability showed coefficients of variation ranging from 0.6% to 4.5%, while the inter-assay variability showed coefficients of variation ranging from 0.7% to 5.2%.

### Statistical Analysis

Statistical analysis was performed using SPSS version 22. Categorical variables were expressed as frequencies and proportions. Continuous variables were presented as mean and standard deviations. Independent *t* test was employed to analyze mean differences between 2 quantitative variables, whereas ANOVA was utilized to assess multiple groups, with the least significant difference (LSD) serving as the post hoc test. *P* value of less than .05 was deemed statistically significant.

## Results

A total of 1000 patients with primary hypothyroidism on stable LT4 doses and in a euthyroid state for ≥ 6 months, out of whom 720 met the inclusion criteria while 280 were excluded (88 patients with hypothyroidism resulting from thyroidectomy, 24 patients with radioiodine ablation, 106 patients with medical conditions or on medications known to interfere with thyroid hormone absorption, and 62 patients with incomplete data). The study included a total of 720 patients, of whom 89.6% (626) were female. The mean age for the study population was 41.37 ± 12.50 years with a mean duration of disease of 12.2 ± 2.8 years. Among the female subjects, 76.5% were premenopausal, and 23.5% were postmenopausal. The majority of subjects were overweight (42.4%) or obese (31.1%). There is no statistically significant difference in mean TSH values across groups over time and gender distribution.

The mean LT4 dose was 99.61 ± 21.42 µg/day. The mean LT4 dose adjusted for actual body weight (LT4/ABW) was 1.51 ± 0.35 µg/kg/day, for ideal body weight (LT4/IBW) was 2.09 ± 0.48 µg/kg/day, and for lean body mass (LT4/LBM) was 2.36 ± 0.51 µg/kg/day.

A comparison of mean LT4 dose requirements for different BMI groups is given in Table 1. The LT4 dose for ABW was significantly less in the higher BMI group as compared to the lower BMI group (*P *< .001) and for IBW, the LT4 dose significantly increased with an increase in the BMI. However, LBM showed no significant change in LT4 across all BMI ranges. A comparison of mean LT4 dose requirements for different age groups is given in Table 2. For different age groups, LT4 dose significantly reduced with advanced age (*P* < .001) in ABW-based dosing but no significant dose change was observed in LBM-based (*P *= .224) and IBW-based (*P *= .377) dosing. A comparison of the mean LT4 dose requirement in premenopausal and postmenopausal female subjects is given in Table 3. ABW-based dosing showed a significant (*P* < .001) lesser dose requirement in postmenopausal patients, but there was no significant difference in the dose observed in IBW- and LBM-based dosing.

**Table 1.** LT4 dosage comparison across BMI groups

BMI group	No of patients	Median age	Mean TSH	Female	Male	LT4/ABW (µg/kg/day)	LT4/IBW (µg/kg/day)	LT4/LBM (µg/kg/day)	*P* value
18.5-24.9 kg/m^2^	191	32.0	2.05 ± 1.22	168	23	1.73 ± 0.34	1.92 ± 0.40	2.37 ± 0.48	<.001*
25-29.9 kg/m^2^	305	41.0	2.05 ± 1.22	253	52	1.51 ± .030	2.09 ± 0.47	2.37 ± 0.52	<.001*
≥30 kg/m^2^	224	44.0	2.47 ± 1.30	205	19	1.33 ± 0.31	2.25 ± 0.51	2.35 ± 0.54	.916

Abbreviations: ABW, actual body weight; BMI, body mass index; IBW, ideal body weight; LBM, lean body mass; LT4, levothyroxine; TSH, thyrotropin (thyroid stimulating hormone).

**Table 2.** LT4 dosage comparison across age groups

Age group	No of patients	Median BMI	Mean TSH (mIU/L)	Female	Male	LT4/ABW (µg/kg/day)	LT4/IBW (µg/kg/day)	LT4/LBM (µg/kg/day)	*P* value
18-45 years	467	26.8	2.3 ± 1.1	412	55	1.56 ± 0.36	2.08 ± 0.47	2.38 ± 0.50	<.001*
46-65 years	227	29.2	2.2 ± 1.3	192	35	1.44 ± 0.33	2.13 ± 0.51	2.34 ± 0.53	.377
>65 years	26	28.1	2.4 ± 1.2	22	4	1.39 ± 0.32	2.02 ± 0.49	2.23 ± 0.52	.224

Abbreviations: ABW, actual body weight; BMI, body mass index; IBW, ideal body weight; LBM, lean body mass; LT4, levothyroxine; TSH, thyrotropin (thyroid stimulating hormone).

**Table 3.** LT4 dosage comparison between premenopausal vs postmenopausal females

Total women (626)	Menstrual status		*P* value
	Premenopausal women (479)	Postmenopausal women (147)	
LT4/ABW (µg/kg/day)	1.55 ± 0.36	1.43 ± 0.31	<.001*
LT4/IBW (µg/kg/day)	2.13 ± 0.48	2.18 ± 0.46	.320
LT4/LBM (µg/kg/day)	2.43 ± 0.50	2.39 ± 0.48	.378

Abbreviations: ABW, actual body weight; BMI, body mass index; IBW, ideal body weight; LBM, lean body mass; LT4, levothyroxine.

BMI was significantly different in different age groups (BMI values were 27.11 ± 4.79, 29.69 ± 4.74, 29.11 ± 4.69 for 18-45 years, for 46-65 and > 65 years respectively, *P* < .001) as well as for premenopausal and postmenopausal (BMI values were 27.56 ± 5.01 and 29.92 ± 4.69 for premenopausal and postmenopausal females respectively, *P* < .001) patients. Multivariate analysis was done for the predictors of ABW-based LT4 dose is shown in Table 4. It showed only BMI was an independent predictor of LT4 dose in ABW-based dosing.

**Table 4.** Univariate and multivariate analyses of levothyroxine dose per actual body weight, with body weight, age, and menstrual status as independent variables

Univariate analysis		Daily LT4 dose/kg of actual BW			
N = 720	β	Coefficients	*P* value	95.0% CI (2 SD)	
				Lower bound	Upper bound
Age	−.006	−0.003	.926	−0.131	0.120
BMI	.981	0.225	<.001*	0.671	1.292
Menstrual status	−.702	−0.014	.719	−4.539	3.135

Multivariate analysis		Daily LT4 dose/kg of actual BW			
N = 720	β	Coefficients	*P* value	95.0% CI (2 SD)	
				Lower bound	Upper bound
Age	−.002	−0.001	.983	−.210	.205
BMI	1.095	0.266	<.001*	.765	1.424
Menstrual status	−3.244	−0.066	.283	−9.177	2.690

Abbreviations: BMI, body mass index; BW, body weight; LT4, levothyroxine.

## Discussion

In our study, we evaluated the factors affecting the LT4 dose and the appropriate method of LT4 replacement in patients with long-standing primary hypothyroidism. IBW-based dosing showed uniform dosage across age groups and menopausal status but showed significant variation across BMI subgroups. LBM-based dosing showed uniform dosage across the different ages, BMI categories, and menopausal status. We found that an LT4 dose of 2.3 µg/kg of LBM was appropriate for all age groups, different BMI groups, and menopausal status. ABW-based dosing showed significant variation in LT4 dosage across the different ages, BMI groups, and menopausal status. Multivariate analysis showed that BMI was an independent predictor of LT4 dosage based on ABW.

### ABW-Based Dosing

In our study, we found that ABW-based dosing was not an appropriate method of LT4 replacement in patients with primary hypothyroidism. The results were comparable with previous published literature [3, 6, 7, 10-13]. Even multivariate analysis in our study showed that BMI was an independent predictor of dose in ABW-based dosing. The appropriate dose in obese individuals is challenging to determine depending on ABW. Using actual body weight for dosing in obese patients may result in overtreatment, while LBM appears to be the most reliable predictor of LT4 needs. This is because key processes in thyroid hormone metabolism, such as deiodination (conversion of T4 to T3), occur predominantly in muscle, liver, and skin tissue rather than adipose tissue [7]. Thus, the use of ABW-based dosing of LT4 does not appear to be physiologically appropriate, especially in individuals with higher BMI.

### LBM-Based Dosing

In our study, we have shown that LBM-based dosing is appropriate for all age groups, BMI groups, and menopausal status, which is comparable with published studies [7, 11, 14]. LBM is crucial for metabolic functions since it consists of body cell mass, extracellular fluid, and non-fatty intercellular connective tissue. The pharmacokinetics of different drugs is mainly determined by LBM as compared to ABW [15]. Hence, LBM-based dosing seems to be the most mechanistically appropriate method to determine LT4 doses. According to our study results, LBM-based dosing of 2.3 µg/kg will be appropriate across all BMI, age, and menopausal groups of patients.

### IBW-Based Dosing

In our study, IBW-based dosing showed no significant variation across patient age groups and menopausal status, but doses were significantly higher in patients with higher BMI. This can be explained by the difference in anthropometric calculation of the IBW and LBM. IBW is based only on height and not on the ABW, whereas the calculation of LBM takes into consideration both height as well as ABW [8]. So, the use of LBM for the calculation of LT4 dose is more appropriate compared to IBW and ABW.

The strengths of our study are that the study focused on long-standing primary hypothyroidism, predominantly caused by Hashimoto thyroiditis, the most prevalent etiology of primary hypothyroidism in iodine-sufficient areas. This is in contrast to prior research that has mostly included post-thyroidectomy hypothyroid patients [13, 16-18]. It is among the few studies exploring LBM-based and IBW-based LT4 dosing across different age groups, BMI, and menopausal status. The large sample size provides robust statistical power. The limitations of our study are that LBM was estimated using a formula (Boer's formula) rather than direct measurement methods like dual-energy x-ray absorptiometry (DXA), which, while more accurate, are impractical for routine use and have additional cost concerns. The use of formulae is more clinically feasible, especially in resource-limited settings, and requires only body weight, height, and gender as data points, and can be easily calculated using online calculators in a few minutes. Another limitation of our study is its cross-sectional nature; prospective validation through randomized controlled trials is needed to confirm our results.

The findings of our study were cross-validated using 3 established equations for LBM estimation: Hume's formula, Kulkarni's formula, and Weij's formula [19-21]. The results obtained using Hume's and Weij's formulas showed concordance with our primary study outcomes. However, application of the Kulkarni formula revealed statistically significant variations in the LT4 dose per kilogram of LBM across different age groups, BMI categories, and menopausal status (for details, refer to Tables S1-S3) [22]. As the Kulkarni formula showed substantial variation in LT4 dose, we recommend using the Boer and Hume formulas for LBM estimation and LT4 dose calculation in routine clinical practice.

## Conclusion

LBM-based dosing is an appropriate method for the LT4 dose calculation in patients of overt primary hypothyroidism as compared to ABW-based and IBW-based dosing. A daily LT4 dose of 2.3 µg/kg of LBM appears to be effective across varying BMI, age groups, and menopausal status. The LBM-based dosing approach is both practical and easy to implement in clinical practice.

## Disclosures

The authors have nothing to disclose.

## Data Availability

Some or all datasets generated during and/or analyzed during the current study are not publicly available but are available from the corresponding author on reasonable request.

## Abbreviations

ABW: actual body weight
BMI: body mass index
IBW: ideal body weight
LBM: lean body mass
LT4: levothyroxine
TSH: thyrotropin (thyroid stimulating hormone)
